# Coding joint: kappa-deleting recombination excision circle ratio and B cell activating factor level: predicting juvenile dermatomyositis rituximab response, a proof-of-concept study

**DOI:** 10.1186/s41927-022-00265-z

**Published:** 2022-05-09

**Authors:** Elisa Ochfeld, Victoria Hans, Wil Marin, Najah Ahsan, Gabrielle Morgan, Lauren M. Pachman, Amer Khojah

**Affiliations:** 1 Pediatric Allergy-Immunology, Ann & Robert H. Lurie Children’s Hospital of Chicago, 225 East Chicago Avenue, Box #60, Chicago, IL 60611 USA; 2 Division of Allergy-Immunology, Department of Pediatrics, Northwestern University Feinberg School of Medicine, Chicago, IL USA; 3 Cure JM Center of Excellence, Stanley Manne Research Center, Chicago, IL USA; 4 Division of Pediatric Rheumatology, Ann & Robert H. Lurie Children’s Hospital of Chicago, Chicago, IL USA; 5 Pediatric Department, College of Medicine, Umm Al-Qura University, Makkah, Saudi Arabia

**Keywords:** Juvenile dermatomyositis, Rituximab response, BAFF level, KREC evaluation

## Abstract

**Background:**

This pilot study’s primary aim was to determine if oligoclonal B cell expansion in children with Juvenile Dermatomyositis (JDM) predicts response to Rituximab therapy. We evaluated: (1) tissue B cell depletion efficacy by measuring the ratio of Coding joint (CJ) to Kappa-deleting recombination excision circle (KREC) DNA, and (2) serum BAFF level upon B cell recovery.

**Methods:**

CJ and KREC values were measured via qPCR assessment of serial PBMC stored (− 80 °C) in the CureJM Center’s BioRepository. Serum BAFF was quantitated by Mesoscale® technology. Oligoclonal B cell expansion was defined as a CJ:KREC ≥ 8 prior to Rituximab therapy. Detection of a CJ:KREC ratio ≤ 2.5 in the first sample after Rituximab was designated as adequate B cell depletion. A significant clinical response to therapy was defined as improvement in Disease Activity Score (DAS) by at least 2 points on consecutive visits within the first 12 months of therapy.

**Results:**

Six out of nine children with JDM showed oligoclonal B cell expansion prior to Rituximab (CJ:KREC ≥ 8). Of those 6 patients, 4 had evidence of effective B cell depletion after Rituximab (CJ:KREC ≤ 2.5), and all 4 of those subjects displayed a significant clinical response to Rituximab. Serum BAFF level increased in 8/9 children after Rituximab.

**Conclusions:**

In this proof-of-concept study, JDM patients with oligoclonal B cell expansion prior to Rituximab have more favorable clinical outcomes after Rituximab. We speculate: (1) B cell depletion post-Rituximab predicts JDM clinical response; (2) increased BAFF post-Rituximab may contribute to disease flare.

**Supplementary Information:**

The online version contains supplementary material available at 10.1186/s41927-022-00265-z.

## Background

Juvenile Dermatomyositis (JDM) is a rare pediatric inflammatory myopathy that involves a characteristic rash and symmetrical proximal muscle weakness [1]. In the majority of JDM cases, the B cell plays an important role in the pathophysiology of the disease evident by B cell infiltration in the muscle tissue and the presence of myositis-specific antibodies (MSA) in patients’ peripheral blood [2, 3]. Rituximab, a chimeric monoclonal antibody directed against CD20 (a protein expressed on the surface of B cells), accomplishes B cell depletion via complement fixation, antibody-dependent cellular cytotoxicity (ADCC), and signaling of apoptosis [4]. Rituximab is utilized to treat Juvenile Dermatomyositis with variable success. This variability is presumed to be linked to the effectiveness of tissue B cell depletion in these patients. While one randomized controlled trial of Rituximab in patients with inflammatory myopathy (RIM trial) did not achieve its primary endpoint, 83% of patients who received Rituximab met the definitions for improvement set by the trial [5]. Post-study analysis from this RCT suggested that specific myositis-specific-autoantibodies (MSAs) including anti-Jo-1 and anti-Mi-2, and young age (JDM vs. adult myositis) predicted better response to Rituximab [6]. Of note, anti-NMDA5 and anti-HMGCoA were not measured in the RIM trial; therefore, patients with these autoantibodies may have been included in the MSA negative group. Currently, Rituximab is reserved for JDM patients who have failed first-line therapy [6, 7]. Markers to help predict which patients will respond best to individual therapies are urgently needed.

CD20 is expressed in the majority of B cell developmental stages, from pre-B cells to memory B cells. These cell types are therefore depleted by Rituximab. CD20 is expressed on B cells until the terminal differentiation of a B cell into a plasma cell and is not expressed on pro-B cells, plasmablasts or plasma cells [8, 9]. These cells are spared during Rituximab treatment, which allows for recovery of the B-cell arm of the immune system after therapy [10]. B cell depletion is variable after Rituximab, but is typically transient, though the duration of time to recovery is unpredictable.

**Fig. 1:**
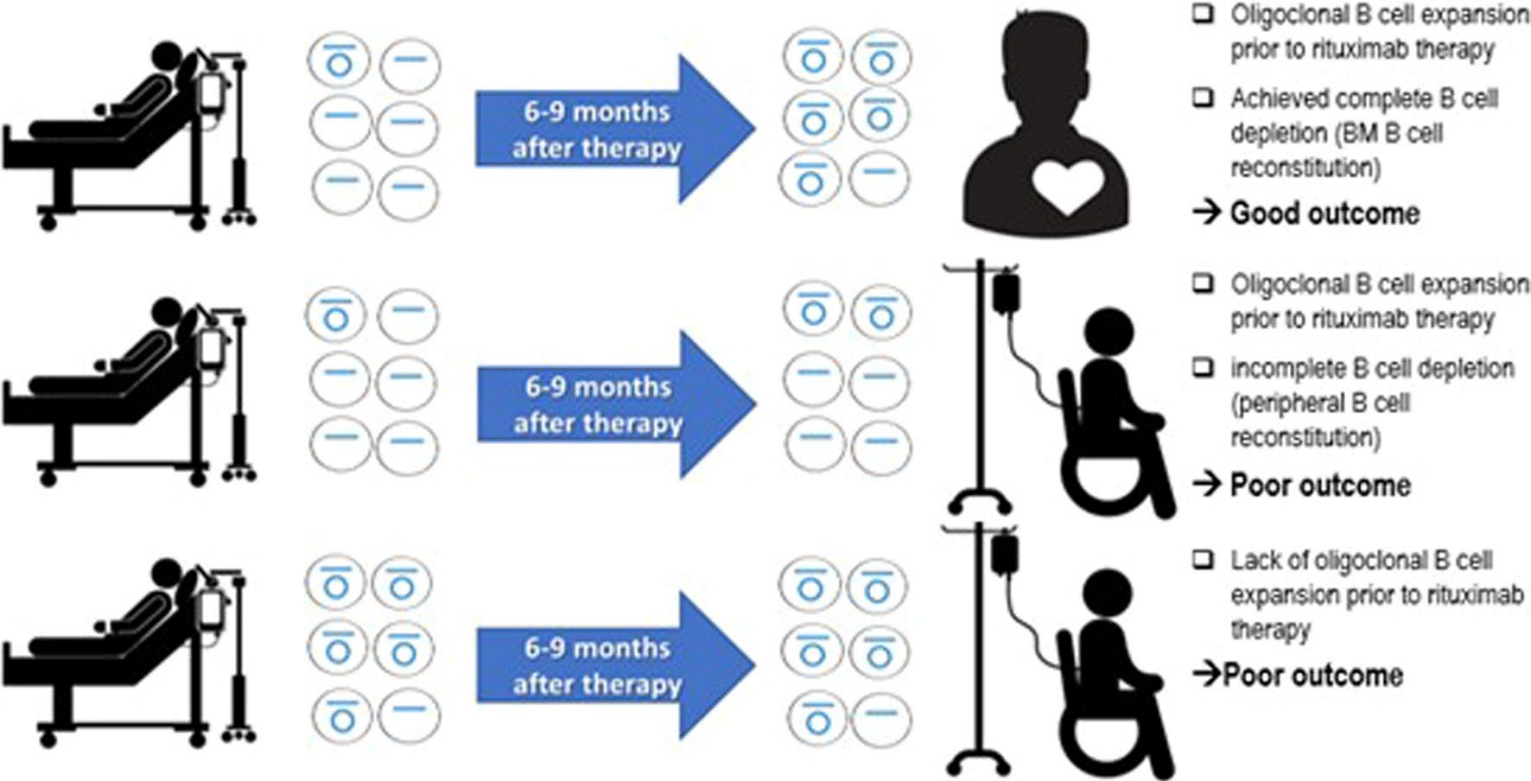
Study hypothesis illustration. JDM subjects with significant oligoclonal B cell expansion will have a good response to Rituximab if they achieved complete B cell depletion evident by bone marrow B cell reconstitution (Image was created by the authors)

The formation of new B cells requires B cell receptor (BCR) V(D)J recombination and involves production of a signal joint. The signal joint is a small unique DNA sequence at the recombination site on the Kappa-deleting recombination excision circle (KREC) once it gets separated from the rest B cell genome. KRECs are small circular excision products *that do not replicate* during B cell division. Therefore, the KREC remains only in one B cell and not in that cell’s progeny [11]. The recombination site on chromosomal DNA is known as the coding joint (CJ), and is found in *all B cell progeny* [11]. The ratio of CJ to KREC DNA, determined by real-time quantitative PCR (qPCR), can be used to estimate the number of B cell divisions that have occurred in a patient’s B cell population [11]. Therefore, an elevated ratio of CJ to KREC can signify oligoclonal B cell expansion which is a hallmark early of B cell immune response to infection or autoimmunity. B Cell Activating Factor (BAFF) is a cytokine produced by activated macrophages and dendritic cells that promotes B cell survival and proliferation. Serum BAFF level is typically elevated in patients with autoimmune diseases such as JDM [12]. The primary aim of this proof-of-concept study is to determine if oligoclonal B cell expansion documented by an elevated ratio of CJ to KREC (before treatment) predicts response to Rituximab therapy. The secondary aim is to evaluate the effectiveness of tissue B cell depletion by measuring the CJ:KREC ratio and serum BAFF level upon B cell recovery. We hypothesize that JDM patients treated with Rituximab who have near-complete tissue B cell depletion display a low CJ:KREC ratio and an elevated soluble serum BAFF level, which will be associated with a more effective clinical response to Rituximab (Fig. 1).

## Methods

After obtaining approval from the Ann & Robert H. Lurie Children’s Hospital of Chicago Institutional Review Board, (IRB 2015-333) a retrospective pilot study was performed at this single freestanding academic children’s hospital. Children under age 21 with JDM were eligible to participate in the study. Data were extracted from the electronic medical record, including sex, race, age at Rituximab infusion, age at JDM onset, number of Rituximab doses, autoantibody profile, treatment regimens, clinical manifestations of JDM and disease activity score (DAS). The DAS is a clinical instrument used to evaluate the perceived activity of skin rash and muscle weakness in children with JDM with good reliability [13].

Coding joint and KREC DNA were quantified using real-time quantitative PCR from serial PBMCs stored at − 80 °C in the CureJM Center Repository. DNA was extracted from PBMCs using a standardized protocol. The study protocol was adapted from prior research from van Zelm et al. [11]. Real-time quantitative PCR assays were designed for the detection of the coding joints and signal joints [11]. Primers and probes amplified the intronRSS-Kde rearrangements (coding joints) and corresponding signal joints (KRECs). The qPCR mixture contained 12.5 µL of TaqMan Universal MasterMix (Applied Biosystems), 2 µL of sample DNA (50 nmol), 2.5 µL of forward primer, 2.5 µL of reverse primer, 1 µL of FAM- TAMRA labeled probe, and 4.5 µL of DI RNAse/ DNAse free water. This gave a total volume per well of 25 µL. This was run on an Applied Biosystems 7500 Fast Real-Time PCR system. To ensure reliability, we ran the samples and healthy controls in triplicates. We also stored all DNA samples in a − 80 °C freezer and re-ran all the samples at the same time in 2 separate 96-well plates. Due to the lack of commercially available predetermined standard samples, we use 2 control samples to generate a standard curve and included them in each run. The C_T_ values of coding joint and signal joint were compared for each sample. As reported by van Zelm et al., the ΔC_T_ (C_T KREC_ − C_T CODING JOINT_) of the qPCRs from a given cell fraction represents the mean number of cell divisions a B cell has undergone [11]. Using the formula ^2^logΔCT, van Zelm demonstrated that this is equal to the coding/ signal joint ratio (CJ:KREC ratio), which can be utilized as a measure for the in vivo replication history of a B cell subset, and therefore a marker of B cell expansion. This approach was validated with in vitro proliferation studies [11]. Serum soluble BAFF levels (pg/mL) were measured using Mesoscale® technology.

Oligoclonal B cell expansion prior to Rituximab therapy was defined as a CJ:KREC ratio of greater than or equal to 8 during the JDM flare-up necessitating rituximab therapy, ideally within 3–6 months before rituximab therapy. Because rituximab approval by medical insurance can take 1–2 months, most of these patients received more immunosuppressive therapy including high-dose IV steroids to treat the disease flare-up pending the insurance approval which may lower their CJ:KREC ratio on the lab assessment on the few weeks preceding IV rituximab. We chose greater than or equal to 8 as the cutoff point for oligoclonal B cell expansion because it represents 3 division cycles and all age matches healthy controls (5 subjects) that we tested had a level below 8 with a mean CJ:KREC ratio of 5 (Additional file 1: Table S1). After Rituximab, a CJ:KREC ratio of less than or equal to 2.5 on the first detectable sample post- Rituximab was considered evidence of effective B cell depletion. Significant clinical response to therapy was defined as improvement of the Disease Activity Score (DAS) by at least 2 points in two consecutive visits after Rituximab.

The software utilized to perform the statistical analysis included PRISM and SPSS.

## Results

Nine JDM patients were included in this study. Demographic characteristics are shown in Table 1. Subjects were 66% female (n = 6), 33% male (n = 3). The mean age at diagnosis of JDM was 4.3 years old. The mean age at first Rituximab dose was 12.55 ± 2.2 years. The majority of subjects were Caucasian (n = 6). Myositis- specific- autoantibodies (MSAs), prior JDM therapy, and disease manifestations are also shown in Table 1. The most common MSA in our population was antibody to p1550/140 (TIF 1-γ—transcriptional factor-1-γ), present in 8/9 patients. All patients had previously been treated with glucocorticoids, 8/9 had previously received cyclosporine, 7/9 had been given IVIG. At the time of study enrollment, months prior to receiving Rituximab therapy, 8/9 patients had cutaneous involvement and 6/9 patients had muscle involvement.

**Table 1:** Clinical and laboratory data pertaining to the 9 children with JDM who were treated with Rituximab

Patient ID	Gender	Race	MSA/MAA	Medication	Age range at 1st Ritux (yr)	No of Ritux doses	Disease Manifestations	Oligoclonal B cell expansion (CJ:KREC ratio)	B cell depletion (CJ:KREC ratio)	Response	Relapse
1	F	W	Ro ind, p155/140 +	CYC, IVIG, MTX, MMF, steroid	10–14	5	S + Mu	Yes (18.2)	Yes (1.5)	Yes	Yes
2	M	W	p155/140 +	CYC, IVIG, MTX, MMF, steroid	10–14	2	S + Mu	Yes (26.5)	Yes (1.5)	Yes	Yes
3	M	W	U1RNP + , MDA5 + , Ro +	MTX, MMF, HCQ, steroid	10–14	1	S only	Yes (8.5)	Yes (1.8)	Yes	No
4	F	H	p155/140 +	CYC, IVIG, MTX, MMF, HCQ, steroid	15–18	1	S only	Yes (18.3)	No (3.9)	No	n/a
5	F	W	p155/140 +	CYC, IVIG, MTX, MMF, HCQ, steroid	10–14	1	S + Mu	Yes (12.8)	Yes (2.4)	Yes	No
6	M	B	MJ +	CYC, MMF, HCQ, steroid	15–18	2	Mu only	Yes (8.7)	No (3.7)	Yes	Yes
7	F	W	p155/140 +	CYC, IVIG, MTX, MMF, HCQ, steroid	10–14	1	S + Mu	No (4.2)	Yes (1.9)	No	n/a
8	F	H	p155/140 +	CYC, IVIG, MTX, MMF, HCQ, steroid	10–14	3	S + Mu	No (3.5)	No (2.6)	No	n/a
9	F	W	p155/140 +	CYC, IVIG, MTX, MMF, HCQ, steroid	15–18	4	S only	No (6.1)	Yes (2.0)	No	n/a

F = Female, M = Male, W = White, H = Hispanic, B = Black, yr = years, S = Skin, Mu = Muscle, CYC = Cyclosporine, MMF = mycophenolate mofetil, IVIG = Intravenous Immunoglobulin, MTX = methotrexate, HCQ = hydroxychloroquine, Ritux = Rituximab

**Fig. 2:**
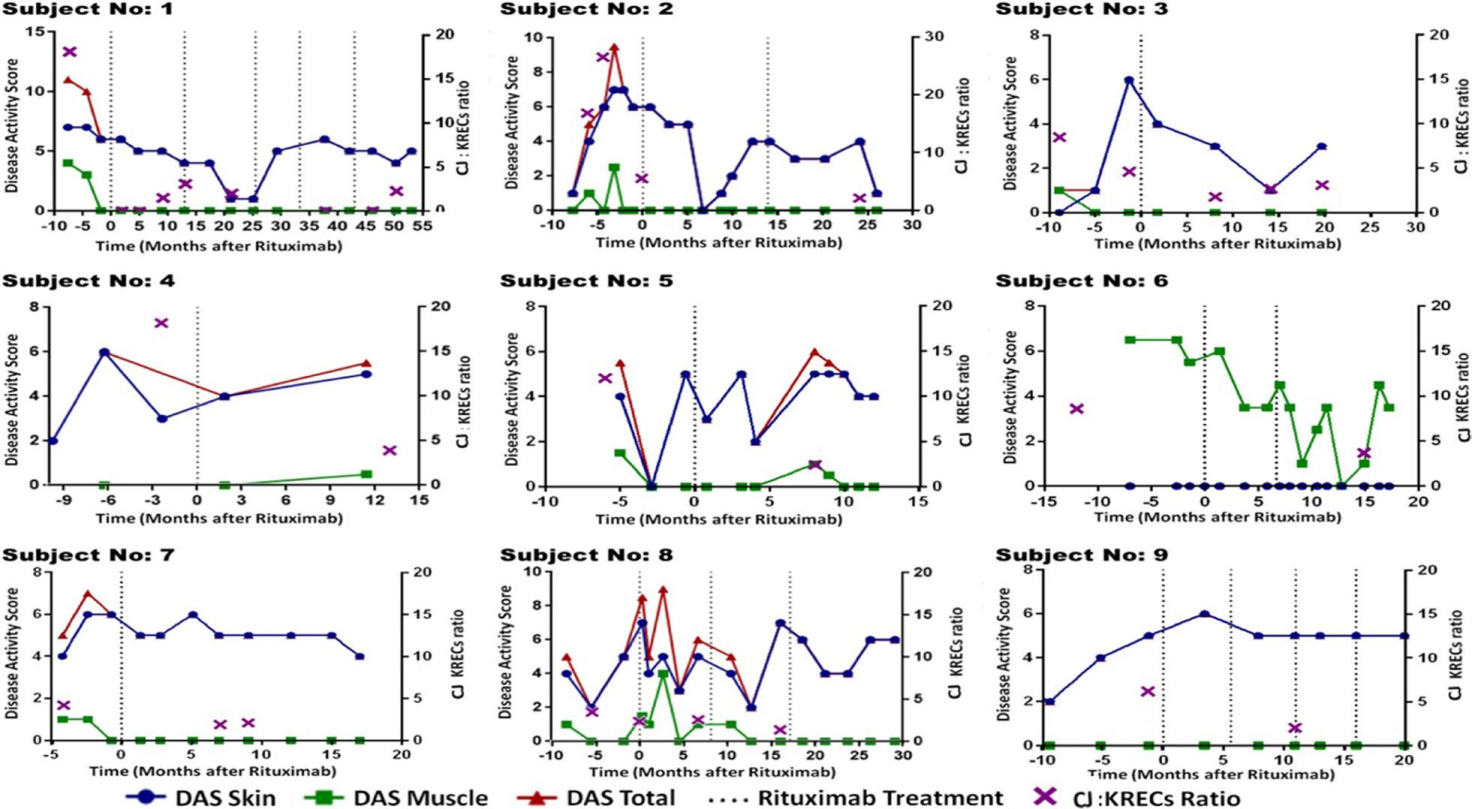
JDM disease course and CJ:KREC ratio. JDM patient response to Rituximab measured by Disease Activity Score (DAS) and its relation to CJ:KREC ratio

Six out of nine children had evidence of oligoclonal B cell expansion prior to Rituximab therapy (CJ:KREC ≥ 8) (Table 1). Of those 6 subjects, 4 had evidence of effective B cell depletion after Rituximab (CJ:KREC ≤ 2.5), and all 4 of those subjects displayed a significant clinical response to Rituximab therapy (improvement of DAS score by at least 2 points within the first 12 months after Rituximab). Regarding the 2 patients who had oligoclonal expansion prior to therapy but did not display effective B cell depletion after Rituximab (CJ:KREC ≥ 2.5), only one showed a significant clinical improvement, but that patient went on to experience a JDM relapse later in their clinical course. Of all patients without effective B cell depletion from Rituximab (n = 3), 2/3 did not show a significant clinical response to therapy, and the 1/3 that did respond clinically then experienced a JDM relapse. All JDM patients without oligoclonal B cell expansion prior to Rituximab had a poor clinical response to Rituximab therapy, regardless of B cell depletion status (Table 1). Figure 2 shows DAS scores, timing of Rituximab therapy, and CJ: KREC evaluation by subject.

**Fig. 3:**
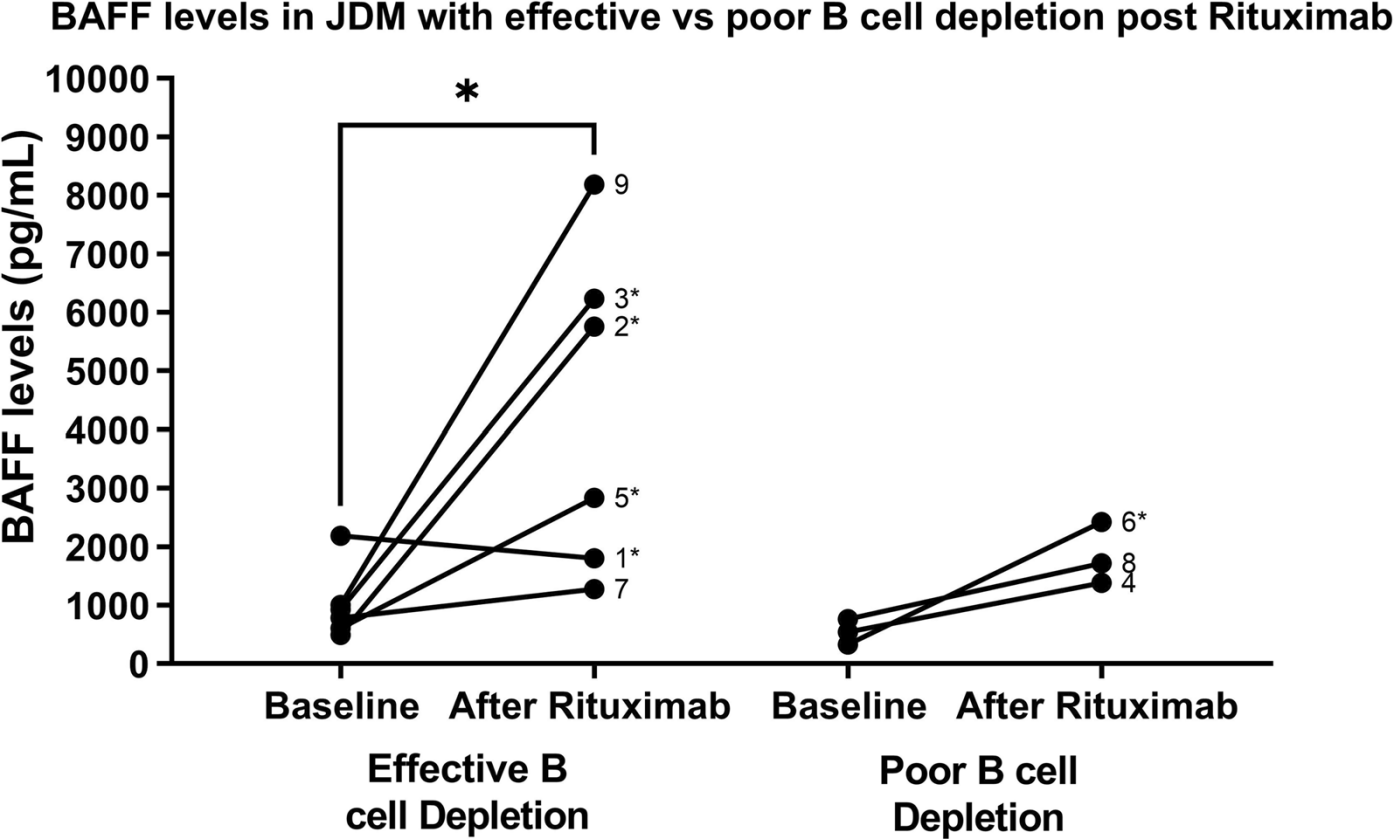
BAFF levels before and after rituximab. BAFF level increased in almost all subjects after Rituximab (8/9). Subjects with poor B cell depletion (CJ:KREC ≥ 2.5) after therapy had only minor increases in serum BAFF level, compared to those with effective B cell depletion

Serum soluble BAFF level increased in almost all subjects after Rituximab (8/9). Subjects with poor B cell depletion (CJ:KREC ≥ 2.5) after therapy had only minor increases in serum BAFF level, compared to those with effective B cell depletion (p = 0.042, with a mean difference of 3,348 pg/mL) (Fig. 3).

## Discussion

Children with JDM who demonstrate oligoclonal B cell expansion (CJ:KREC ≥ 8) prior to the use of Rituximab have more favorable clinical outcomes after Rituximab therapy. If this finding is confirmed by subsequent studies, the CJ:KREC ratio may serve as a selection criterion for Rituximab therapy in JDM patients. Additionally, qPCR analysis of Coding joint (CJ) and KREC DNA can replace more expensive laboratory evaluations (such as flow cytometry of B cells and B cell subsets), and can be utilized to predict response to therapy and monitor B cell reconstitution. This evaluation could be utilized in a wide variety of clinical settings including international and/ or limited health resource settings, to help predict response to therapy and guide medication management when flow cytometric measures are not available.

Prior research has attempted to identify predictors of clinical improvement in myositis patients treated with Rituximab. Anti-synthetase and anti-Mi-2 MSAs in JDM appear to be associated with lower disease damage and increased clinical improvement in patients with refractory myositis [7]. According to current guidelines, Rituximab is reserved for JDM patients who have failed first-line therapy [6, 7]. Precision medicine approaches to complex patients with refractory JDM are needed, and we propose that the CJ:KREC ratio can assist in the identification of JDM patients who are most likely to respond to B cell depleting therapy.

Serum soluble BAFF level generally increases after Rituximab due to B cell depletion as a mechanism to promote B cell count recovery. However, in patients with poor B cell depletion, there is only a minor increase in serum BAFF level after treatment. Patients with X- linked Agammaglobulinemia (who have absent B cells due to a genetic defect) have significantly higher soluble BAFF concentrations compared to healthy controls (p < 0.001) [14]. Our data, taken together with prior research, suggests that the more significant the B cell deficit, either iatrogenically induced or due to an inherited B cell deficiency, the higher the resulting soluble BAFF level. Further research is required to understand if this increase in serum BAFF level after B cell depletion contributes to disease flare post-Rituximab, and if so, it could potentially provide an additional therapeutic target in JDM. Belimumab is a humanized IgG1γ antibody directed against BAFF, currently approved for the treatment of systemic lupus erythematosus (SLE). Directed therapies such as this could be important disease modulators in other autoimmune diseases such as JDM.

The limitations of this study include the small numbers of subjects, which requires validation in additional cohorts. Additionally, this was a single-center retrospective review, thus its generalizability may be limited. We did not assess adult myositis patients, thus age differences in response to Rituximab therapy cannot be extrapolated from this work. Finally, there is no commercially available predetermined standard sample to calibrate KREC and JC RT-PCR assay. Creating such a sample will facilitate wider adoption of this technology and assure test reliability between different laboratories.

## Conclusions

Based on this proof- of concept study, Juvenile dermatomyositis patients with oligoclonal B cell expansion prior to Rituximab therapy likely have more favorable clinical outcomes after Rituximab therapy however larger study is needed to verify this finding. We speculate that the degree of B cell depletion after Rituximab predicts JDM clinical response and that increased BAFF post-Rituximab may contribute to disease flare.

## Supplementary Information

**Additional file 1.** Demographic data and CJ:KREC ratio from healthy children.

## Data Availability

The data that support the findings of this study are available from the corresponding author, [AK], upon reasonable request.

## Abbreviations

JDM: Juvenile Dermatomyositis
CJ: Coding joint
KREC: Kappa-deleting recombination excision circle
BAFF: B-cell activating factor
qPCR: Quantitative polymerase chain reaction
DAS: Disease Activity Score
MSA: Myositis-specific antibodies
ADCC: Antibody-dependent cellular cytotoxicity
RCT: Randomized controlled trial
BCR: B cell receptor
TIF 1-γ: Transcriptional factor-1-γ
