# Supplementary material for: Coding joint: kappa-deleting recombination excision circle ratio and B cell activating factor level: predicting juvenile dermatomyositis rituximab response, a proof-of-concept study
Source: BMC Rheumatol. 2022 May 9;6:36. doi: 10.1186/s41927-022-00265-z (PMC9082850; doi:10.1186/s41927-022-00265-z)
Supplement: Supplementary file 1 — Additional file 1. Demographic data and CJ:KREC ratio from healthy children. [file 41927_2022_265_MOESM1_ESM.docx]

**Supplemental table 1: CJ:KREC ratio in healthy controls**

| ID | Sex | Age range | CJ:KREC ratio |
| --- | --- | --- | --- |
| 1 | Female | 5-9 years | 6.9 |
| 2 | Male | 10-14 years | 5.2 |
| 3 | Female | 5-9 years | 2.5 |
| 4 | Male | 15-18 years | 2.0 |
| 5 | Female | 10-14 years | 7.6 |
